# Biomonitoring Pilot Surveys of Zearalenone in Breastmilk and the Urine of Children in Central Portugal

**DOI:** 10.3390/toxins17040162

**Published:** 2025-03-25

**Authors:** Sofia Duarte, Inês Duarte, Myrella Duarte, Ana Paiva, Ricardo Cabeças, Liliana J. G. Silva, André M. P. T. Pereira, Celeste Lino, Angelina Pena

**Affiliations:** 1 Centro de Investigação Vasco da Gama (CIVG)/Departamento de Ciências Veterinárias, Escola Universitária Vasco da Gama (EUVG), Campus Universitário, Av. José R. Sousa Fernandes, 3020–210 Coimbra, Portugal; ana.paiva@euvg.pt (A.P.); ricardo.cabecas@euvg.pt (R.C.); 2 LAQV, REQUIMTE, Laboratório de Bromatologia e Farmacognosia, Faculdade de Farmácia da Universidade de Coimbra, Polo III, Azinhaga de Sta Comba, 3000–548 Coimbra, Portugal; ines.f.duarte@hotmail.com (I.D.); myrellamfalves@gmail.com (M.D.); ljgsilva@ff.uc.pt (L.J.G.S.); andrepereira@ff.uc.pt (A.M.P.T.P.); clino@ff.uc.pt (C.L.); apena@ci.uc.pt (A.P.)

**Keywords:** mycotoxin, milk, breastfeeding, contaminants, exposure, risk

## Abstract

Zearalenone (ZEA) is a mycotoxin that acts primarily as an endocrine disruptor. Biomonitoring studies are needed to assess exposure and risk, particularly among vulnerable groups. This study reports two pilot biomonitoring surveys of ZEA in 38 lactating mothers and 42 children (5–12 years old). Both were associated with a questionnaire to collect data on the sociodemographics and eating habits of the participants. About 76% of urine samples were contaminated (188.12 ± 235.99 ng/mL), with the hazard quotient reaching 2.36 in the worst-case scenario for younger children. Of the analyzed breastmilk samples, 55.26% were contaminated (158.26 ± 77.50). A statistically significant association between ZEA contamination of breastmilk and the maternal consumption of wholemeal bread, cereal flakes, sausages, smoked meat and pork was found, suggesting that these foods are determinants of higher exposure. The hazard quotient in the worst-case scenario for breastfed babies under 16 weeks was estimated as 0.61. Results confirm frequent exposure to this endocrine disruptor among these two vulnerable groups in central Portugal, showing the need for further studies.

## 1. Introduction

Zearalenone (ZEA) is one major mycotoxin biosynthesized by filamentous fungi of the genus *Fusarium*, namely *F. graminearum*, *F. culmorum*, *F. cerealis*, *F. equiseti*, *F. crookwellerse*, *F. semitectum*, *F. verticillioides*, *F. sporotrichioides*, *F. oxysporum* and *F. acuminatum* [1]. This field mycotoxin accumulates in cereal crops, mainly corn, but also barley, oats, rice, sorghum, rye and wheat [1,2]. The highest frequencies and levels of ZEA were reported in maize/corn and feed mixtures used for fattening pigs. Grain-based products, like baked goods, pasta, breakfast cereals and bread are also frequently reported to be contaminated [1].

ZEA is mostly excreted through feces and urine [3], but it has also been detected in milk, including in breastmilk [4,5,6]. The elimination of ZEA by mammals via the enterohepatic route may result in the reabsorption of ZEA glucuronides destined for excretion through bile, promoting the prolonged retention of ZEA and its metabolites in the bloodstream; this consequently extends the duration of the resulting toxic effects [3,7]. A range of modified forms of ZEA have been detected in food and feed, with several such metabolites exhibiting endocrine activity that could, in some cases, exceed that of ZEA [8]. Endocrine disruption in animals and humans results from the chemical similarity between their chemical non-steroidal structures and the chemical structures of endogenous estrogens like 17-β-estradiol [9]. Exposure to ZEA and its metabolites is associated with precocious puberty and premature development of the breasts [10,11].

According to the International Agency for Research on Cancer (IARC) [12], ZEA is included in group 3, that is, this mycotoxin is not classifiable as carcinogenic to humans. ZEA has low acute toxicity, but prolonged exposure can carry health risks [3]. ZEA is also considered an immunomodulator and immunotoxin, with hepatotoxic effects [1]. Additionally, in the scope of the public-health impact, the contribution of ZEA to the disease burden of non-communicable diseases should not be neglected [13].

Pregnant women and children are among the most vulnerable population groups in relation to exposure to mycotoxins. Generally, biotransformation of xenobiotics is slower in newborns than in adults, which results in greater absorption of chemicals ingested through the diet [14]. Babies are more vulnerable to the adverse effects of mycotoxins compared to adults due to the immaturity of their immune system, lower detoxification capacity, lower body mass and higher metabolic rate. Based on the above, the assessment of exposure to mycotoxins in children is of great importance [15]. EFSA concluded that ZEA exposure does not vary in relation to gender; however, it was found that the youngest age group (children and adolescents) has greater exposure to ZEA compared to adults and elderly people; this was explained by younger people’s consumption of greater volumes of food per kg of body weight [16].

Its toxic properties and the difficulty associated with preventing its production and with decontamination contribute to the seriousness of the risk that ZEA poses to public health and food safety. As critically reviewed by [8], given that current assessments of ZEA-associated health risks and exposure suffer from major uncertainties and methodological limitations, human biomonitoring (HBM) is a useful, more reliable approach. HBM studies allow a direct approach to exposure assessment and make it possible to overcome the main limitations of the indirect approach, namely the heterogeneous distribution of mycotoxins in food [17] and the inaccuracy of consumption data provided by participants [18]. HBM considers the various sources and routes of exposure (oral, dermal and inhalation) [19] in the evaluation process, as well as the individual characteristics and lifestyle habits of each individual [20]. This monitoring technique further allows the identification of patterns of exposure to the contaminants [20].

Given the above, this HBM study aimed at the assessment of ZEA exposure and the associated risk in breastfed infants and children. The study also aimed to identify determinants of exposure in terms of anthropometric and socio-demographic factors, as well as eating habits. This is the first reported study of ZEA occurrence in breastmilk in Portugal, to the authors’ knowledge.

## 2. Results

### 2.1. Urine Biomonitoring Study

Of the 42 urine samples analyzed, 32 (76.19%) showed ZEA levels higher than the LOD (50 ng/L), with an average concentration of 0.19 ± 0.24 μg/L and a maximum value of 1.28 μg/L.

Considering the variables retrieved from the questionnaire (Table 1), it is notable that the frequency of contamination grows as the educational levels of the parents increase. Regarding the gender of the children, boys showed the highest frequency of contamination and the highest mean level. Young children and, accordingly, children with the lowest body weights, presented with the highest mean and maximum ZEA levels. Children with higher consumption of homemade/locally produced products presented with the highest mean and maximum ZEA levels.

Except for ice cream (*p* = 0.03), the present study did not find any statistically significant correlation between urinary ZEA levels and the exposure variables studied (*p* > 0.05).

As shown in Table 2, the worst scenario for the group aged between five and eight years presents hazard quotient (HQ) values greater than one, representing a risk to public health. Nevertheless, in all age groups, considering average levels of ZEA (i.e., average-case scenario), HQ ranges from 0.02 and 0.53, meaning that the estimated PDI is thirteen to two times lower than the established tolerable daily intake (TDI; 250 ng/kg bw/day) [16].

### 2.2. Breastmilk Biomonitoring Study

The survey of the 38 breastmilk samples detected 21 positives, which means that more than half of the samples (55.26%) were contaminated with ZEA levels above the detection limit (60 ng/L). The average level was 158.26 ± 77.50 ng/L, up to a maximum level of 341.42 ng/L.

As shown in Table 3, most of the positive samples (42.86%) as well as the highest average ZEA level (180.92 ± 84.79 ng/L), were obtained from mothers between 30 and 33 years old. Most of the mothers with ZEA-contaminated breastmilk had a high-school education level (42.86%), with the sample in which the maximum level of ZEA was detected coming from that group. Primiparous mothers produced milk with higher average and maximum levels of ZEA.

The greatest number of positive samples corresponded to samples collected during the winter season (42.86%), although the contamination levels in these samples were lower than the levels in samples collected during the summer. The majority of contaminated breastmilk samples (42.86%) were consumed by babies younger than 3 months old, although the oldest babies (older than 7 and 11 months old) consumed milk with higher average ZEA levels.

The greatest proportion of breastmilk samples (42.86%) were collected from mothers who reported eating food mostly acquired from supermarkets (75%). The highest mean ZEA levels were found in breastmilk samples from mothers who reported that half of the food they consumed was from supermarkets (175.91 ± 86.06 ng/L).

The Wilcoxon test revealed statistically significant associations between the consumption of wholemeal bread (*p* = 0.04), cereal flakes (*p* = 0.03) and sausages and smoked meat (*p* = 0.05) and the contamination of breastmilk. With an association close to significance, it was found that the consumption of pork (*p* = 0.09) contributed to the occurrence of positive samples.

As presented in Table 4, the ZEA exposure and associated risk were higher for the youngest babies (one to three months old), particularly in the worst-case scenario, considering the maximum level of ZEA detected in the breastmilk of the corresponding mothers. Nevertheless, in none of the scenarios studied did the estimated daily intake (EDI) surpass the TDI (250 ng/kg bw/day) [16]. The scenario in which the HQ value was higher was the worst-case scenario for the group of babies between 1 and 3 months of age. It is noteworthy that in this group, the TDI considered was that adjusted for babies under 16 weeks (83 ng/kg bw/day), as recommended by EFSA [15,24].

## 3. Discussion

### 3.1. Urine Biomonitoring Study

Published studies related to ZEA exposure in children are scarce. Compared to the few previous similar studies in children, the average concentration in the present study (0.19 ± 0.24 μg/L) was higher than the average concentration recorded in the United Kingdom (0.09 μg/L) [25] and in Belgium [26], where ZEA was not detected in the samples. In the Austrian 2020 children’s biomonitoring survey, Ayeni et al. [27] reported that ZEA was detected in all analyzed urine samples (85 boys and girls, aged 6–10 years), up to a maximum level of 0.59 μg/L, with an average level of 0.14 μg/L.

In comparison with the studies carried out in the adult population of Spain (6.7 ± 7 μg/L) [28]; Transquei, South Africa (0.20 ± 0. 46 μg/L) [29]; Austria (0.39 μg/L) [21]; Nigeria (0.3 μg/L) [30] and Portugal (1.30 μg/L) [17], the present study showed lower contamination levels of ZEA in the urine of the sampled children. In contrast, the levels of ZEA found in the sampled children in the present study were higher than those detected in the adult populations of Haiti [31] (<0.15 μg/L), Sweden (0.09 ± 0.07 μg/L [32], Germany (0.03 ± 0.02 μg/L) [33] and Tunisia (<0.03 μg/L) [34], who were less exposed to ZEA than the population of children analyzed in the present study. These results can be explained by the fact that younger people consume a greater volume of food per kg of body weight compared to adults [35]. The physiological vulnerability of children justifies the inclusion of children as a group at risk from the toxic effects of contaminants. Despite the low acute toxicity, long-term exposure to ZEA is concerning given its potent estrogenic activity, which may result in a health risk [8]. The reproductive and developmental toxicity of ZEA observed in experimental and farm animals [16] raises further concern when analyzing the results of the exposure in children. Despite having scarcely been studied, the potential combinatory effects of ZEA and its modified forms with other co-occurring food xenobiotics can result in an additional risk to human health and so should not be neglected [8]. Mixtures of chemicals found in food, encompassing synthetic (e.g., bisphenol A) and natural (e.g., phytoestrogens) endocrine disruptors, along with endogenous estrogens, contribute to total human exposure and may influence the estrogenicity of ZEA [8,11].

In the analysis of parents’ educational levels, it was found that as the parents’ educational level increases, the ZEA content also increases, even if the effect is not statistically significant (see Table 1). None of the studies carried out previously conducted an analysis according to the educational level of the parents.

Boys presented with a higher frequency of samples with detectable ZEA content (62.5%), a higher average ZEA content (0.20 ± 0.29 μg/L) and a higher maximum value detected (1.28 μg/L). By contrast, females have the lowest minimum value (0.06 μg/L) (see Table 1). Of the four studies carried out only in children, only Gratz et al. [25] related ZEA levels to gender, but they did not find a statistically significant difference between girls and boys. Among the studies whose study population included children and adults, only three found this relationship. Föllmann et al. [33] described higher average ZEA levels in males, a finding similar to that of the present study. Contrary to this finding, Li et al. [36] and Wallin et al. [32] described higher values in females. In the specific case of girls, it appears that the average concentration of positive samples in the present study (0.18 ± 0.11 μg/L) was above the average concentration reported in Turkey (0.08 ± 0.06 μg/L) [37] and below the average concentration reported in New Jersey (1.82 ± 4.80 μg/L) [38].

In relation to the age of children, it was found that the concentration of ZEA decreases with increasing age (see Table 1).

Regarding body weight, the highest average content (0.32 ± 0.44 μg/L) and the highest maximum value (1.28 μg/L) were found in the group of children weighing between 18 and 29 kg (see Table 1). It was observed that ZEA content decreases with increasing weight. This result can be explained by the fact that children consume a greater volume of food per kg of body weight.

It was found that, regarding the origin of the food products consumed, the ZEA content increases with an increase in the percentage of homemade products or local products and with a decrease in the percentage of products purchased in commercial areas (see Table 1). A possible explanation may be that operators of companies in the food and feed sectors are aware of the presence of mycotoxins and therefore must comply with current legislation and implement measures to prevent and control mycotoxins, unlike individuals who produce food for their own consumption.

Apart from ice cream, the present study did not find any statistically significant correlation between urinary ZEA levels and the exposure variables studied (*p* > 0.05). In the literature, there are few studies that investigate the presence of ZEA in ice cream. Zhang et al. [39] detected ZEA in a sample of xanthan gum (6 ± 0.6 ng/g), which can be used as an additive to improve the consistency of ice creams and other dairy products, among other products. Researchers detected the presence of ZEA in flour (levels between 1 and 24 μg/kg), which is an ingredient in cake-batter ice cream [40]. The occurrence of ZEA in ice cream may originate from the use of potentially contaminated milk. Flores-Flores et al. [41] brought together studies on the occurrence of ZEA in bovine milk samples in Hungary, the United Kingdom, Egypt and China, reporting levels of up to 12.5 μg/kg. In China, Huang et al. [42] quantified ZEA levels in bovine milk of up to 20.5 ng/kg.

It is noteworthy that in a recent multi-mycotoxin-exposure study in UK children (29 boys and girls aged 2.4–6.8 years), even though ZEA was the fourth-most-frequently detected mycotoxin (48.2% of ZEA positive samples), it was not associated with the intake of any of the food groups considered [43].

Due to the lack of consensus on the urinary excretion rate of ZEA in humans [35], to assess the risk, different exposure scenarios were determined, considering the minimum, average and maximum content of ZEA detected in the urine, along with different excretion rates reported in the literature, and using the average body weight of participating children in each age group (see Table 2). It is important to underline that assessment of internal exposure based on ZEA excreted in urine presents uncertainties given the scarcity of data on the urinary excretion rate of ZEA in humans [35]. Excretion rates depend on how ZEA and its metabolites are excreted, in free or conjugated form, but also on the species, age and sex of the study population [44]. Thus, estimates of exposure to ZEA obtained through analysis of urine should be regarded as an approximation, given the lack of representative and large-scale studies on human ZEA-excretion patterns [45]. Accordingly, additional studies are necessary regarding the toxicokinetic profile of ZEA in humans [22].

For all age groups, the HQ values, in the best-case scenario, are between 0.01 and 0.22, regardless of the excretion rate considered (Table 2). Even though they do not represent a risk according to the HQ approach [17], the estimated risk should not be neglected for two reasons: first, children are a vulnerable group; second, as underlined by Ayeni et al. [27], the facts that mixture effects of multiclass contaminants are not yet considered in health-based guidance values and that ZEA can be further degraded into more potent active metabolites should raise concern.

### 3.2. Breastmilk Biomonitoring Study

This was the first reported biomonitoring survey of ZEA in breastmilk in Portugal. The frequency of contamination observed in the breastmilk analyzed in the current study (55.26%) was lower than those reported previously in Angola (100%) [4], Turkey (100%) [6] and Italy (100%) [5]. However, the value was slightly higher than that reported in Spain (37%) [46]. The mean ZEA level detected in the breast milk samples analyzed was close to the median value reported in a similar study in Turkey (173.8 ng/L) [6]. However, it should be noted that only the maximum value determined in the present study (341.42 ng/L) is close to the mean value reported in previous studies in Angola (380.7 ± 256.7 ng/L) [4] and Italy (1130 ± 340 ng/L) [47].

The differences in the reported frequency and mean level of ZEA contamination between studies could result from differences in the performance of the analytical methods employed. Furthermore, the differences reported could also result from variations in environmental conditions during the pre-harvest stage, from inappropriate storage conditions, and from food-consumption patterns specific to each country or region [16].

Like in the urine biomonitoring study, higher education levels were associated with a higher frequency of contamination; education was also associated with a higher maximum level (high-school education) and average level (university education). By contrast, in the Angolan study, the highest mean and maximum levels were found in mothers with only an elementary-level education (408.0 ± 301.7 (72.5–1487.4) ng/L).

In line with the findings from the breastmilk samples analyzed in Angola [4], the highest mean ZEA levels were observed in younger (up to 33 years old; 436 ± 360 (185.2–1487.4)) and primiparous mothers (612.0 ± 318.0 (166.2–1077.9)). Nevertheless, like in previous studies [4,6], no statistically significant correlation was found between these variables and ZEA levels in the analyzed breastmilk.

The only parameters from the food questionnaire that showed a positive direct correlation with ZEA levels in breastmilk were those regarding consumption of some of the food groups, like wholemeal bread (*p* = 0.04) and cereal flakes (*p* = 0.03). Previously, in the study conducted in Angola, a statistically significant association was found between ZEA contamination and biscuit consumption (*p* = 0.0003) [4]. Cereal-derived food products are recognized to be more susceptible to ZEA contamination, explaining why maximum levels of ZEA are set only for cereals and cereal-derived foods within the regulatory framework of the European Union [48]. Furthermore, grains and grain-based foods have already been previously identified by EFSA [16] as the largest contributors to zearalenone exposure in all age classes. In the study period, RASFF issued only two notifications related to ZEA in cereals and cereal-derived foods: the first for organic rice crackers from Belgium and the second for wheat from the Czech Republic [49].

It is important to notice that cereals are major components of the feed of farm animals, but in the current regulatory framework of the European Union, no maximum levels of ZEA are imposed for products intended for animal feed materials or complementary and complete feeds; instead, the framework only makes recommendations [50]. In this respect, it is noteworthy that in the present study, consumption of sausages and smoked meat (*p* = 0.05) and, with a finding close to statistical significance, pork (*p* = 0.09) was associated with higher ZEA levels. Previously, a Turkish study [36] also found a statistical correlation between ZEA breastmilk levels and meat. Swine are considered the most susceptible animal species for ZEA [15], and the first reported case of natural ZEA contamination of feed, in 1928, was reported for swine feed [51]. Recently, Ropejko and Twarużek [1] observed that had at least 77% of samples of corn, beans, grains and feed mixtures intended for fattening pigs were contaminated by ZEA, based on worldwide reported data. Regarding ZEA content, samples of corn, corn grains, fibrous animal feed and feed mixtures for fattening pigs and fish feed had the highest levels of ZEA content. These data confirm the susceptibility of grains and animal feed to ZEA exposure [1]. Nonetheless, reported occurrences of ZEA in pork are scarce.

## 4. Final Remarks

Despite the importance of the results of the surveys, this study was impacted by recognized limitations, namely in the study design (cross-sectional), limited size of the sample populations, type of analysis performed (screening through ELISA) and the poor quality of information available from previous studies on the urinary excretion of ZEA.

Nevertheless, the present study contributes evidence of frequent exposure of two vulnerable groups to ZEA, a finding scarcely reported in the literature. In addition, among exposure determinants, educational level was clearly associated with higher ZEA exposure. Younger babies and children, who have lower body weights, were also found to have higher ZEA exposure. While ZEA-contaminated urine was associated with higher consumption of ice cream by children, ZEA-contaminated breastmilk was associated with maternal consumption of wholemeal bread, cereal flakes, sausages, smoked meat and pork.

Further, broader biomonitoring studies are essential to provide the required data for a comprehensive assessment of human-health risks related to dietary exposure to ZEA.

## 5. Materials and Methods

### 5.1. Sampling

This cross-sectional study was approved by the Scientific Council of the Faculty of Pharmacy of the University of Coimbra and the Ethics and Animal Welfare Committee of the Vasco da Gama University School (EUVG) (Opinion no 2019/001) and respected the applicable legal framework. All participants (the parents/legal guardians in the case of children) were informed about the objective and methodologies of the study and about the guarantee of confidentiality under Law 58/2019, of 8 August.

The biological (convenience) samples were collected in the central region of Portugal. The 42 urine samples were collected in 2022 from children between five and 12 years of age, and the 38 breastmilk samples were collected between 2018 and 2022 from lactating women.

The **urine** samples were collected via the children’s voluntary urination, independently or with assistance from the guardian. The first urine of the morning was collected after discarding the first stream; the samples were then refrigerated and protected from light. Once in the laboratory, they were immediately frozen until the date of analysis. The inclusion criteria were that the children be healthy and between 5 and 12 years old, with an informed-consent statement signed by the parent/legal guardian and a completed questionnaire.

The **breastmilk** samples were collected by the participants themselves after they had received instructions on hygiene and storage. They were stored in sterile bags suitable for breast milk, frozen and kept frozen at −20 °C until analysis. The inclusion criteria defined for the mothers participating in the study were as follows: being healthy and over 18 years old, with the birth having occurred more than one month previously to avoid the inclusion of colostrum or transitional milk.

### 5.2. Sociodemographic Data and Eating Habits

To identify potential patterns and determinants of ZEA exposure, on the day of sample collection, each participant (or their parent/legal guardian in the case of the urine) completed a questionnaire consisting of three parts: I-Anthropometric data and individual characteristics; II-Sociodemographic data; and III-Data related to nutrition.

The anthropometric data considered were the children’s sex, age, weight and height, as well as whether any medication had been taken in the week before collection and, in this case, what medication had been taken. In the case of lactating women, the data collected were profession, number of children, date of the birth, the baby’s birth weight and the baby’s weight at the time of milk collection.

The sociodemographic data considered were related to the residence and the educational level and profession of the participant in the case of lactating women and of the parents in the case of the children.

Additionally, a semi-quantitative food-frequency questionnaire regarding the participant’s consumption habits in the seven days prior to sample collection was filled out. The food groups included in this part of the questionnaire were dairy products, breads and cereals, eggs, meat and fish, vegetables, sweets and nuts.

### 5.3. Analytical Determination of ZEA

In accordance with the manufacturer’s instructions, the urine samples were hydrolyzed before analysis. For this purpose, 0.5 mL of urine sample was diluted in 3 mL of 50 mM acetate buffer solution (pH 4.8). In each test tube, 8 μL of glucuronidase/arylsulfatase solution from *Helix promatia* (Merck, Reference: 4114) was also added, and then the solutions were shaken and incubated for 3 h at 37 °C.

After incubation, in the case of urine samples, the next step was purification of the hydrolyzed urine using RIDA^®^ C18 columns (R-Biopharm AG^®^, Darmstadt, Germany) at a flow rate of 1 drop per second. The columns were washed with 3 mL of methanol (100%) and equilibrated with 2 mL of 20 mM Tris buffer solution pH 8.5/methanol in a ratio of 80/20 (*v*/*v*). Then, 3.5 mL of hydrolyzed urine was poured, and the column was washed with 2 mL 20 mM Tris buffer solution pH 8.5/methanol in the ratio 80/20 (*v*/*v*). The columns were washed with 3 mL of methanol (40%) and dried with nitrogen flow for 1 min. The sample was then eluted slowly (15 drops per minute) with 1 mL of methanol (80%) and the eluate was placed in a water bath at 60 °C to evaporate. Finally, the dry residue was redissolved in 50 μL of methanol and 450 μL of sample dilution buffer and carefully shaken.

The determination of ZEA in the biological samples was performed by the competitive ELISA enzyme immunoassay (R-Biopharm, AG, Darmstadt, Germany), with samples run in duplicate.

The results from the six duplicate ZEA standard solutions in each kit were considered, including both % absorbance and coefficient of variation between replicates. According to the manufacturer, the detection limits (LODs) corresponded to 50 ng/L (for urine samples) and 60 ng/L (for breastmilk).

To determine the ZEA concentration in the biological samples, RIDA^®^SOFT Win software (Reference: Z9999) was applied.

### 5.4. Risk Assessment

The assessment of exposure to ZEA through urine biomonitoring was carried out through estimation of the probable daily intake (PDI) value, as follows: PDI (μgkg/bw/day) = C × V/W × E. In this equation, C corresponds to the ZEA concentration in positive urine samples (in μg/L); V represents the volume of urine excreted by children for 24 h (1 L); W corresponds to the average body weight of the children under study (in kg); and, finally, E corresponds to the excretion rate of ZEA as a percentage [28]. Due to the uncertainty regarding the toxicokinetic profile of this mycotoxin in humans, several scenarios were considered, incorporating the different age/weight groups and two excretion rates retrieved from the literature (9.4% [21] and 36.8% [22,23]).

The assessment of exposure to ZEA through breastmilk biomonitoring was carried out through calculation of the estimated daily intake (EDI) value, as follows: EDI (ng/kg body weight/day) = (C × M)/W. In this equation, C corresponds to ZEA concentration in positive samples (ng/L); M to milk consumption (in L); and W corresponds to the body weight of the children (in kg). The mean body weight was calculated considering the infants’ weight at the time of breastmilk collection. The weight was not available for one child.

For infants weighing up to 7 kg, a daily consumption of milk of 150 mL/kg per day was considered, while for infants who weighed 7 kg or more, 1 L per day was considered. Considering EFSA guidance [15], a consumption of 260 mL/kg bw per day was considered for infants up to 16 weeks of age. For infants older than 16 weeks, a daily consumption of 1 L per day was considered.

For the risk assessment, the tolerable daily intake (TDI), as established by EFSA (250 ng/kg bw/day) [16], was considered.

The TDI was corrected by a factor of three (i.e., 83 ng/kg bw/day) to account for reduced excretory function as well as exclusive breastfeeding in infants under 16 weeks of age [15,24].

The hazard quotient (HQ), which consists of the ratio between PDI (for urine) or EDI (for breastmilk) and TDI, allows for risk assessment. If the HQ is less than one, exposure does not represent a public-health concern [17].

### 5.5. Statistical Analysis

The data were anonymized, edited and categorized in Microsoft ^®^ Excel (Microsoft Office Home and Student version 2016). The preliminary descriptive analysis and the statistical analysis were performed using Microsoft Excel and R statistical software (R 4.2.1) Given the non-normal distribution of the data, the non-parametric Wilcoxon test was used, as this test allows the comparison of two dependent samples. The *p*-value of the test considered was *p* < 0.05.

## Figures and Tables

**Table 1:** Levels (μg/L) in ZEA-positive urine samples according to the variables considered.

Variable		ZEA-Positive Urine Samples		
		n (%)	Mean ± SD (µg/L)	Range (µg/L)
Father’s education level	Elementary education	4 (12.5)	0.09 ± 0.03	(0.08; 0.28)
	High school	21 (65.63)	0.14 ± 0.11	(0.06; 1.28)
	Higher education	7 (21.88)	0.16 ± 0.15	(0.07; 0.47)
	Elementary education	1 (3.13)	0.28	(0.28)
Mother’s education level	High school	14 (43.75)	0.16 ± 0.14	(0.07; 0.47)
	Higher education	17 (53.13)	0.20 ± 0.30	(0.06; 1.28)
Gender	Boy	20 (62.5)	0.20 ± 0.29	(0.06; 1.28)
	Girl	12 (37.5)	0.17 ± 0.11	(0.07; 0.46)
Age (years)	5–8	8 (25)	0.29 ± 0.42	(0.07; 1.28)
	9–10	15 (46.88)	0.16 ± 0.15	(0.06; 0.55)
	11–12	9 (28.13)	0.14 ± 0.08	(0.07; 0.27)
Body weight (kg)	18–29	7 (21.88)	0.32 ± 0.44	(0.66; 1.28)
	30–39	15 (46.88)	0.15 ± 0.11	(0.66; 0.47)
	40–61	10 (31.25)	0.15 ± 0.15	(0.06; 0.55)
Homemade products/local products	0%	4 (12.5)	0.09 ± 0.03	(0.07; 0.12)
	<25%	11 (34.38)	0.15 ± 0.09	(0.06; 0.47)
	50%	12 (37.5)	0.17 ± 0.18	(0.07; 0.55)
	>75%	5 (15.63)	0.36 ± 0.52	(0.06; 1.28)
Products purchased in supermarkets	<25%	4 (12.5)	0.41 ± 0.58	(0.06; 1.28)
	50%	7 (21.88)	0.16 ± 0.17	(0.08; 0.55)
	75%	14 (43.75)	0.17 ± 0.14	(0.06; 0.47)
	100%	7 (21.88)	0.12 ± 0.07	(0.07; 0.28)

**Table 2:** Best, worst and average scenarios for each age group, based on the average body weight of each group.

Age (Mean Weight)	Scenario	ZEA Level (µg/L)	Exposure and Risk Assessment	Excretion Rate (%)	
				9.4 [21]	36.8 [22,23]
5–8 years (23.08 kg)	Best case	minimum level = 0.07 µg/L	PDI (µg/kg bw/day)	0.03	0.01
			HQ	0.13	0.03
	Average	average level = 0.29 µg/L	PDI (µg/kg bw/day)	0.13	0.03
			HQ	0.53	0.14
	Worst case	maximum level = 1.28 µg/L	PDI (µg/kg bw/day)	0.59	0.15
			HQ	2.36	0.60
9–10 years (39.67 kg)	Best case	minimum level = 0.06 µg/L	PDI (µg/kg bw/day)	0.01	0.01
			HQ	0.06	0.02
	Average	average level = 0.16 µg/L	PDI (µg/kg bw/day)	0.04	0.01
			HQ	0.17	0.04
	Worst case	maximum level = 0.55 µg/L	PDI (µg/kg bw/day)	0.15	0.04
			HQ	0.59	0.15
11–12 years (37.55 kg)	Best case	minimum level = 0.07 µg/L	PDI (µg/kg bw/day)	0.02	0.02
			HQ	0.08	0.02
	Average	average level = 0.14 µg/L	PDI (µg/kg bw/day)	0.04	0.01
			HQ	0.16	0.04
	Worst case	maximum level = 0.27 µg/L	PDI (µg/kg bw/day)	0.08	0.02
			HQ	0.31	0.08

**Table 3:** Levels (ng/L) of ZEA in ZEA-positive breastmilk samples according to the variables considered.

Variable		ZEA-Positive Breastmilk Samples		
		n (% of Positives)	Mean ± SD (ng/L)	Range (ng/L)
Mother’s age	26–29 years	3 (14.29%)	155.21 ± 77.27	62.1–304.57
	30–33 years	9 (42.86%)	180.92 ± 84.79	70.76–341.42
	34–37 years	5 (23.81%)	117.22 ± 79.61	77.34–171.01
	38–42 years	4 (19.05%)	160.86 ± 72.25	86.08–245.22
Education level	Elementary school	2 (9.52%)	81.71 ± 76.90	77.34–86.08
	High school	5 (23.81%)	159.28 ± 105.49	70.76–304.57
	University	9 (42.86%)	162.77 ± 77.47	62.1–341.42
	MSc/PhD	5 (23.81%)	179.75 ± 55.69	128.37–235.85
Season of collection	Spring	4 (19.05%)	143.49 ± 77.81	62.1–235.85
	Summer	6 (28.57%)	198.91 ± 79.07	98.96–341.42
	Fall	2 (9.52%)	143.36 ± 62.22	90.41–196.31
	Winter	9 (42.86%)	141.04 ± 84.09	70.76–304.57
Baby’s weight at collection (x^−^ = 7.53 ± 2.05 ng/L)	3.2–5 kg	5 (23.81%)	91.31 ± 75.67	62.1–118.98
	5.1–7 kg	6 (28.57%)	204.43 ± 86.46	77.34–341.42
	7.1–9 kg	5 (23.81%)	160.21 ± 65.71	70.76–245.22
	9.1–11.35 kg	4 (19.05%)	171.05 ± 81.02	80.26–304.57
	No response	1 (4.76%)	155.1	n.a.
Infant’s age (x^−^ = 5.58 ± 4.38 ng/L)	1–3 months	9 (42.86%)	114.30 ± 83.47	62.1–196.31
	4–6 months	6 (28.57%)	77.34 ± 92.46	77.34–341.42
	7–10 months	4 (19.05%)	182.0 ± 77.25	70.76–304.57
	≥11 months	2 (9.52%)	163.1 ± 70.37	155.1–171.01
Number of children (x^−^ = 1.44 ± 0.55 ng/L)	1	9 (42.86%)	188.75 ± 86.36	98.96–341.42
	2	12 (57.14%)	135.39 ± 76.64	62.1–245.22
	3	0	0	0
Homemade products/local products	0	3 (14.29%)	170.76 ± 70.88	70.76–245.22
	≤25%	8 (38.10%)	136.14 ± 81.73	62.1–235.85
	50%	6 (28.57%)	175.91 ± 87.99	90.41–341.42
	75%	4 (19.05%)	166.65 ± 106.18	80.26–304.57
	100%	0	0	0
Products purchased in supermarkets	0	0	0	0
	≤25%	3 (14.29%)	120.67 ± 65.02	80.26–195.68
	50%	6 (28.57%)	175.91 ± 84.06	90.41–341.42
	75%	9 (42.86%)	154.86 ± 95.86	62.1–304.57
	100%	3 (14.29%)	170.76 ± 70.88	70.76–245.22

Note: To assess exposure, we inferred the weight of the baby for whom no weight data were available based on their age.

**Table 4:** Best, worst and average scenarios according to age and weight, based on the average body weight of each group.

Variable		Scenario	ZEA Level	Exposure and Risk Assessment	
Age	1–3 months (i.e., younger than 16 weeks) n = 9 positives x^−^ = 6.01 kg	Best-case scenario	Minimum ZEA level (62.1 ng/L)	EDI (ng/kg bw/day)	16.15
				HQ	0.19
		Average scenario	Average ZEA level (114.30 ng/L)	EDI (ng/kg bw/day)	29.72
				HQ	0.36
		Worst-case scenario	Maximum ZEA level (196.31 ng/L)	EDI (ng/kg bw/day)	51.04
				HQ	0.61
	≥4 months (i.e., older than 16 weeks) n = 12 positives x^−^= 7.83 kg	Best-case scenario	Minimum ZEA level (70.76 ng/L)	EDI (ng/kg bw/day)	9.04
				HQ	0.04
		Average scenario	Average ZEA level (191.23 ng/L)	EDI (ng/kg bw/day)	24.42
				HQ	0.10
		Worst-case scenario	Maximum ZEA level (341.42 ng/L)	EDI (ng/kg bw/day)	43.60
				HQ	0.17
Weight	Breastfed < 7 kg (n = 11 positives) x^−^ = 5.24 kg	Best-case scenario	Minimum ZEA level (62.1 ng/L)	EDI (ng/kg bw/day)	9.32
				HQ	0.04
		Average scenario	Average ZEA level (153.01 ng/L)	EDI (ng/kg bw/day)	22.95
				HQ	0.09
		Worst-case scenario	Maximum ZEA level (341.42 ng/L)	EDI (ng/kg bw/day)	51.21
				HQ	0.20
	Breastfed ≥ 7 kg (n = 9 positives) * x^−^ = 8.78 kg	Best-case scenario	Minimum ZEA level (70.76 ng/L)	EDI (ng/kg bw/day)	8.06
				HQ	0.03
		Average scenario	Average ZEA level (165.03 ng/L)	EDI (ng/kg bw/day)	8.06
				HQ	0.03
		Worst-case scenario	Maximum ZEA level (304.57 ng/L)	EDI (ng/kg bw/day)	8.06
				HQ	0.03

* The baby of the mother with a positive ZEA sample who did not indicate the baby’s weight at the time of collection was not considered; HQ, hazard quotient.

## Data Availability

The original contributions presented in this study are included in the article. Further inquiries can be directed to the corresponding author.
